# Degree of conversion and microhardness of resin cements photoactivated through glass ceramic

**DOI:** 10.4317/jced.58630

**Published:** 2021-11-01

**Authors:** Carolina-Nemesio-de Barros Pereira, Cláudia-Silami Magalhães, Frederico-Santos Lages, Raquel-da Conceição Ferreira, Emerson-Hamilton da Silva, Rodrigo-Richard da Silveira, Elaine-Carballo-Siqueira Corrêa, Cristiano-Leite Fantini, Allyson-Nogueira Moreira

**Affiliations:** 1 PhD, Professor, Department of Restorative Dentistry, Faculty of Dentistry, Federal University of Minas Gerais, Belo Horizonte, Brazil; 2 PhD, Professor, Department of Social and Preventive Dentistry, Faculty of Dentistry, Federal University of Minas Gerais, Belo Horizonte, Brazil; 3 PhD, Private office; 4 PhD, Professor, Department of Metalography, Centro Federal de Educação Tecnológica de Minas Gerais, Belo Horizonte, Minas Gerais, Brazil; 5 PhD, Professor, Department of Optical Physics, Instituto de Ciências Exatas, Federal University of Minas Gerais, Belo Horizonte, Brazil

## Abstract

**Background:**

To assess whether glass-ceramic shade, thickness and translucency affect degree of conversion (DC) and Knoop microhardness (KHN) of resin cements photoactivated using light-emitting diode (LED) or quartz-tungsten-halogen (QTH) units.

**Material and Methods:**

Glass-ceramic blocks were cut (2, 3 and 4mm) and sintered. For DC FT Raman spectroscopy (n=3), film specimens of cements (RelyX ARC, U200, Veneer, C&B) were obtained. For KHN test (n=3), cements were inserted in cylindrical matrix and covered by polyester strip. Specimens were photoactivated (30s) using LED or QTH according to each group: direct photoactivation (DP), interposing ceramic specimens or no photoactivation (NP). Data were analysed by ANOVA and Tukey’s test, Kruskal-Wallis and Dunn’s tests (*p*<0.05).

**Results:**

Ceramic features had significant effect on DC of RelyX ARC, U200 and Veneer (*p*<0.0017). Light source had no effect (*p*=0.9512). C&B and Veneer had higher DC, followed by dual cements. NP dual cements showed the lowest DC. For KHN, ceramic shade (*p*=0.1717) and light source (*p*=0.1421) were not significant, but ceramic translucency, thickness and resin cement were significant (*p*=0.0001). KHN was higher for U200 followed by ARC, and lowest for Veneer.

**Conclusions:**

DC was affected by ceramic shade, translucency and thickness. KHN was dependent on ceramic translucency and thickness. Higher DC and KHN were achieved for dual-cured cements photoactivated through 2mm-thick low translucent or 3mm-thick high translucent glass-ceramic.

** Key words:**Cementation, composite resin cements, dental curing lights, glass ceramics.

## Introduction

Metal-free ceramic restorations have been widely used in dentistry due to their excellent aesthetic, biocompatibility and durability (1-3). Lithium disilicate glass-ceramics have a high mechanical strength and aesthetic besides their capacity of adhesive bonding to resin cements (4) and the possibility of obtaining CAD/CAM (computer-aided-design/computer-aided-manufacturing) restorations (5,6).

Ideally, to promote the stress distribution through the tooth structure, ceramic restorations should be adhesively cemented and the most reliable performance is achieved with light-cured, self-cured or dual-cured resin cements. SuiTable polymerization occurs in the presence of light at the appropriate wavelength and irradiance (3,7,8). Thus, ceramics translucency should not be considered only as an aesthetic parameter but also as a determining factor in polymerization of the underlying resin cement. Adequate resin cement polymerization is also influenced by the chemical composition (3,5) and thickness of the ceramic (9-13).

The influence of the type of light-curing unit on the characteristics of the resin cements polymerization through indirect restorations has been studied (14-16). Whereas the combination of scattering, reflection and absorption may explain the attenuation of the incident light through the ceramic, the interference of underlying resin cement polymerization pattern can compromise the longevity of the restoration (3,15,17).

It was previously shown that light-emitting diode (LED) and quartz-tungsten-halogen (QTH) light transmission through the CAD/CAM lithium disilicate glass-ceramic were both effective, but the halogen source was superior. Concerning the ceramic features, high translucency, low thickness and shades A1 and A2 were associated with higher light transmission (18). The attenuation of the light by ceramic may be compensated considering the concept of the energy density that is the product of the total intensity emitted by the exposure time (14). The more opaque, darker and thicker the ceramic, the greater the exposure time on each face of the restoration is, aiming to provide enough power for the proper polymerization of the underlying resin cement (18).

Given the above, the objective of this study was to evaluate whether lithium disilicate glass-ceramic shade, thickness and translucency affect the degree of conversion (DC) and Knoop microhardness (KHN) of different resin cements photoactivated using LED or QTH units. The null hypothesis is that there is no effect of the shade, thickness, and translucency of the lithium disilicate glass-ceramic or the light source on the DC and the KHN of the resin cements evaluated.

## Material and Methods

In this experimental *in vitro* study, the factors shade (A2, A3 and A3.5), thickness (2, 3 and 4mm) and translucency (high and low) of the IPS e.max CAD ceramic, resin cements (dual conventional RelyX ARC, dual self-adhesive RelyX U200, light cured RelyX Veneer, self-cured C & B), and the light source (Light emitted diode, LED Bluephase and quartz-tungsten-halogen, QTH, Demetron) were investigated. The dependent variables were the percentage of the degree of conversion (DC) and the number of Knoop microhardness (KHN) of resin cements. Considering the 3 levels of the factors thickness, 2 of translucency, 3 of shade, 3 light-sensitive resin cements and 2 light sources, 324 specimens were included (n=3). For the positive controls, with direct photoactivated cement without the interposition of ceramics, 18 samples were used. There were six negative controls of each dual-cured cement without photoactivation, and for the self-cured C&B Cement, three specimens were prepared.

Eighteen e.max CAD ceramic blocks (shades A2, A3 and A3.5, high translucency — HT and low translucency — LT) were cut on a precision cutter (IsoMet® 1000, Buehler, Illinois, USA) 2, 3 and 4 mm-thick and then sintered in accordance with the manufacturer recommendations. For the degree of conversion analysis (n=3), each cement was manipulated in accordance with the manufacturer’s recommendations and was then loaded on a glass slide (24x50mm, Digilab, Piracicaba, SP). A polyester strip (Fava, Sao Paulo, SP) followed by another glass slide were immediately placed on cement, under a load of 30g under a precision scale (Mars A500, São Paulo, SP) producing a film specimen (110 to 190μm). For microhardness analysis (n = 3), the cements were manipulated according to the manufacturer’s recommendations on a paper pad and inserted in a cylindrical polyacetal split matrix (Ø2x1.5mm), covered with a polyester strip. For both tests, the resin cements were photoactivated for 30 seconds using a light-emitting diode (LED Bluephase, 1350mW/cm2, energy density 40.5J/cm2) or quartz-tungsten-halogen (QTH Demetron, 950mW/cm2, energy density 28.4J/cm2) units, according to each group: direct photoactivation (DP); interposing ceramic specimens (experimental groups); or no photoactivation (NP). The NP protocol was not performed for RelyX Veneer, which is only light cured, or C&B, which is self-cured. All specimens were stored dry and protected from light for seven days at 37°C. The cement films were subjected to FT Raman spectroscopy (Vertex RAM II70 Burker, Germany) to evaluate the degree of conversion (19) with a resolution of 4 cm−1 and 32 scans ranging from 3600 to 300 cm−1. The absorption peaks of the aromatic double bonds at 1608 cm−1 and aliphatic double bonds (1636 cm−1) were recorded for each specimen with 60 seconds of integration time of each spectrum (Software OPUS 7.5, CO, UK). The DC was calculated as DC = [1 - (R polymerized/R non-polymerized)] x 100. The cylindrical specimens were subjected to a Knoop microhardness test (50g, 15s, 9 indentations in 3 equidistant parallel lines - Schimadzu HMV2, Japan), which already provides final values of KHN of each indentation. KHN mean for each specimen was calculated. Data analysis (α = 5%) were performed by ANOVA and Tukey’s test for the degree of conversion and the Kruskal-Wallis test, Dunn’s test and Bonferroni correction for Knoop microhardness.

## Results

**Figure 1:**
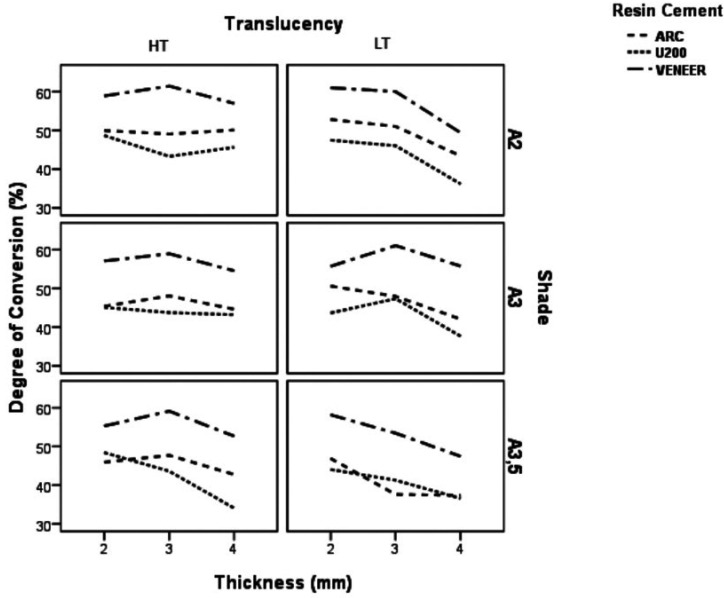
Mean DC (%) for the dual and photoactivated cured cements concerning variation of ceramic thickness, color and color translucency.

The ANOVA showed that, except for the light source (*p* = 0.9512), the other factors were significant for the DC (*p*<0.0017). Figure 1 represents the mean DC of light-cured and dual-cured cements according to ceramic thickness, shade and translucency.

For ARC cement, there was a significant effect of translucency (F=10:49, *p*= 0.001), shade (F=8.10, *p*=0.0001), thickness (F=7.04, *p*=0.014) and the translucency and thickness interaction (F=4.15, *p*=0.0185) on DC (Table 1). For the U200 cement, there was a significant effect of ceramic thickness (F=18.74, *p*=00:00), translucency (F=10.99, *p*=0.0001) and shade (F=3:47, *p*=0.035) on DC (Table 2). The interaction of translucency, thickness and shade was significant (*p*=0.0462). For the RelyX Veneer cement, the effects of ceramic thickness (F=16:51, *p*=0.000), translucency (F=13:48, *p*=0.000) and shade (F=5:56, *p*=0.000) were significant (Table 3). However, no interaction among the factors was significant (*p*> 0.0608).

**Table 1:**
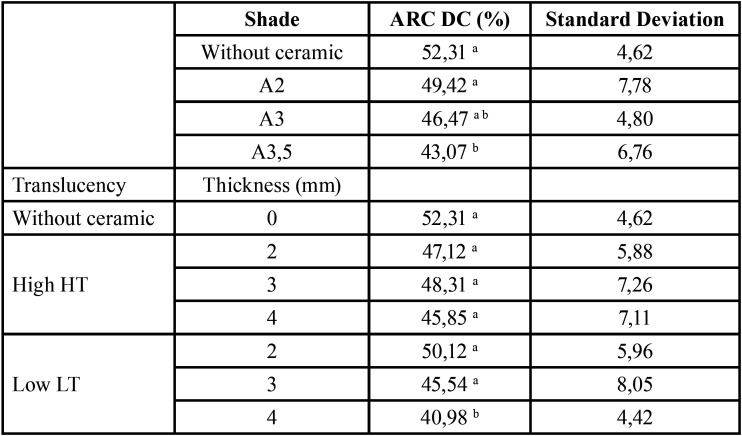
Degree of conversion (DC, mean value) and standard deviation of RelyX ARC considering ceramic shade, and combinations of ceramic translucency and thickness.

**Table 2:**
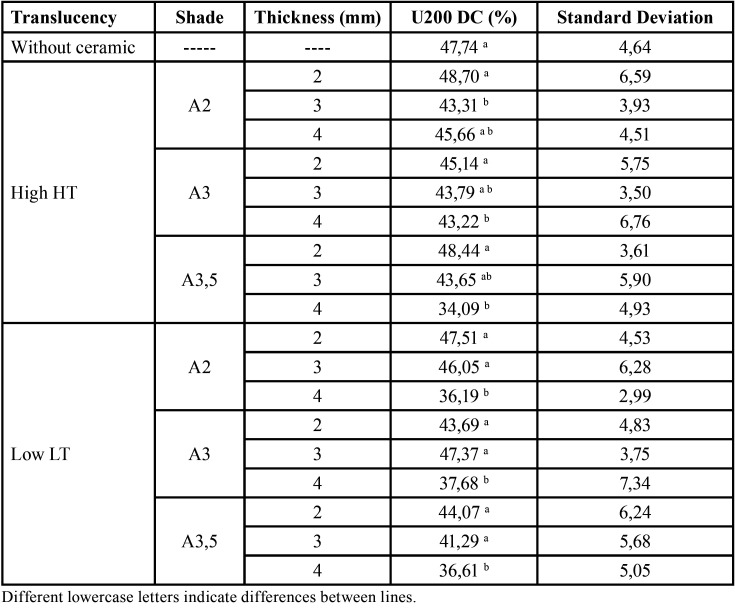
Degree of conversion (DC mean value and standard deviation) of RelyX U200 considering ceramic shade, translucency and thickness.

**Table 3:**
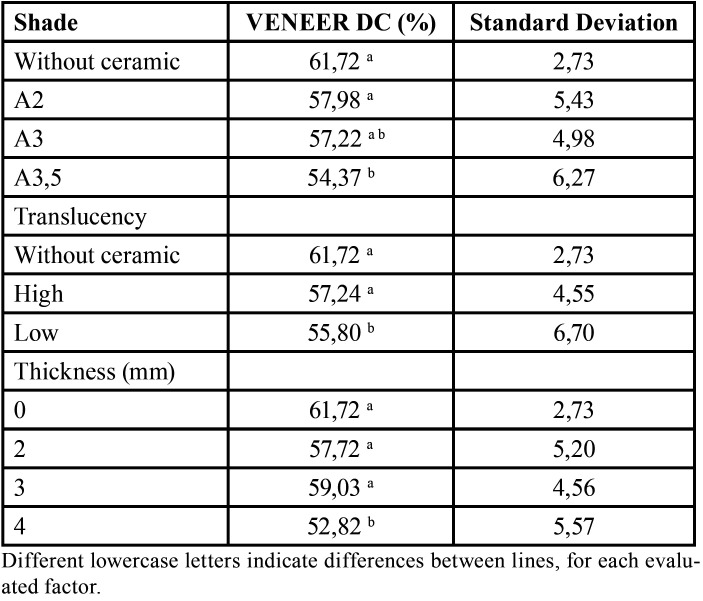
Degree of conversion (DC, mean value) and standard deviation of RelyX Veneer considering ceramic shade, ceramic translucency and ceramic thickness.cency and thickness.

**Figure 2:**
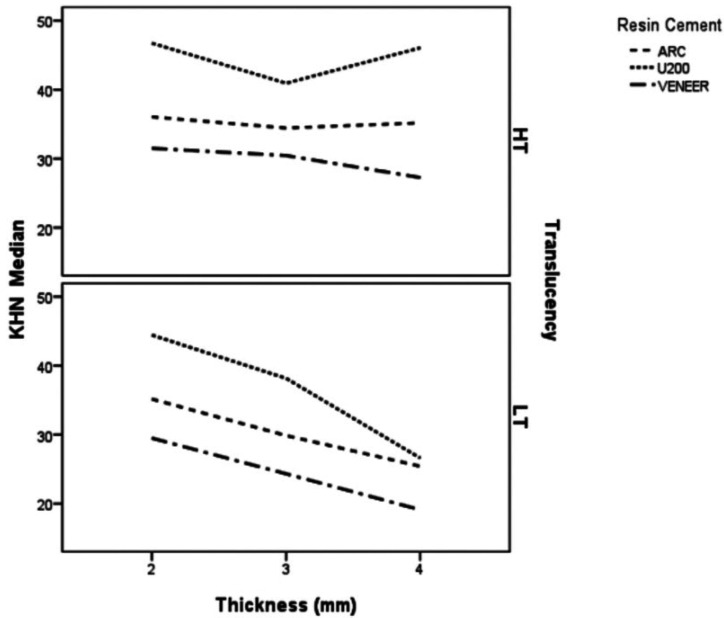
KHN mean of each resin cement in function of ceramic translucency and thickness.

There was no significant effect of ceramic shade on KHN of evaluated cements (*p*=0.1717). There were significant differences in KHN depending on the type of cement and the combination of ceramic thickness and translucency (*p*<0.0001). Table 4 shows the ranking of the KHN values that identifies differences between data. Figure 2 illustrates an inverse relationship between the KHN values and the thickness of low translucency ceramic. High translucency ceramics were associated with higher resin cement KHN values compared to the low translucency.

**Table 4:**
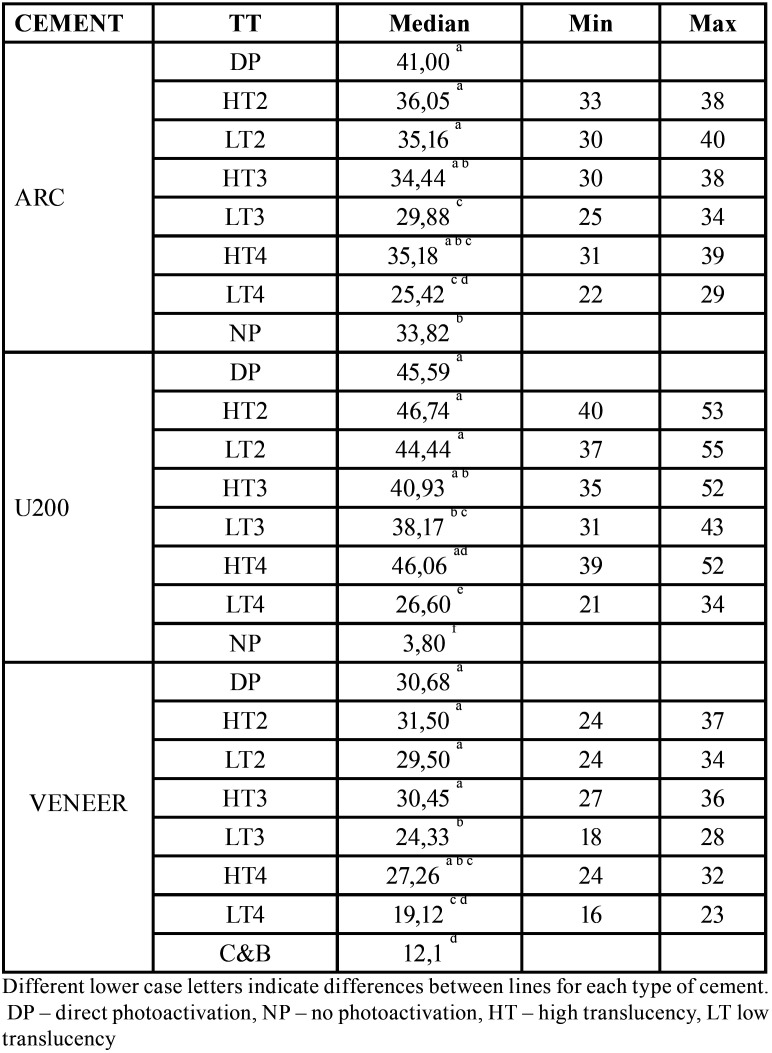
Resin cements KHN median for ceramic translucency and thickness combined (TT).

## Discussion

The null hypothesis that there is no effect of the light source on the degree of conversion and the hardness of evaluated resin cements was accepted, while the null hypothesis that there is no effect of glass-ceramic lithium disilicate shade, translucency and thickness was rejected. For light-cured cement and for self-cured, high DC values were observed, but the hardness values were low. Although hardness is related to the material’s strength, DC would be more representative of its behaviour (10,12,20,21), and a positive correlation between hardness and the DC was found (15,22,23). However, the present study didn’t find this correlation.

The ceramic restorations should be cemented using self-cured cements as they do not depend on light to initiate the polymerization reaction. However, these cements present limitations as short setting time, poor colour stability, high concentration of tertiary amines and postoperative sensitivity (24). In this study, C&B showed a high DC, but the KHN values were the lowest. Ideally, the cement elasticity modulus should be intermediate between the restorative material and the substrate, and low hardness values are associated with a decreased integrity of the adhesive interface (24). Moreover, the reaction between the parts of self-cured resin cement occurs at lower rates than the light-cured or dual cements as free radicals formed by chemical components are entrapped in the polymer network and cannot contribute to an overall increase in the DC and microhardness (14).

However, the irradiation of light-cured or dual-cured resin cements through ceramic decreases the intensity of the light with increasing the thickness and opacity of the ceramic material (8,11,18,25). In this study, the interposition of the ceramic showed that the microhardness was more dependent on the final energy incident on the resin cements than the degree of conversion, as also previously related (26). The ceramic material itself interposed between the light source and the resin cement attenuates the light intensity which can negatively influence the polymerization of the dual resin or light curing cement (13,15,16,20), as also observed in this study. Despite the exponential reduction of light intensity through the ceramic observed previously (18), the reduction of the DC values and KHN of dual and light-cured cements through the same ceramic was not so marked (8). Based in our results, the translucency had a greater impact than the thickness in the DC and KHN.

Ceramic interposition can decrease the microhardness and colour stability of resin cements, especially for thicker and bilayer ceramics, although not always being correlated with the DC (13). Otherwise, increasing the porcelain thickness by up to 1.5 mm has no adverse effect on the degree of conversion of both dual-cure and light-cure resin cements (3). It can be suggested that light-cured cements are more sensitive to light. Although the intensity has been attenuated by the ceramic presence, the light transmitted was sufficient to initiate the polymerization reaction of the RelyX Veneer and ensure high DC values, unlike initially expected. As the KHN values were low, this irradiation couldn’t be sufficient to ensure the formation of crosslinks which occur in a later stage of the polymerization and respond in part by increasing the mechanical properties of the resin cement (12).

For dual cements, a significant reduction of the mechanical properties when cements were photoactivated through lithium disilicate reinforced glass-ceramics from 2mm (9,10,22) or 3mm thickness has been reported (8,11,23,26) and lithium disilicate glass-ceramic shade A2 and 2mm thickness did not affect the DC but decreased the microhardness of the cements. Thus, dual cements are the first choice for cementing thicker restorations because they have a chemical activation of the components (26,27) associated with light activation which ensures clinically acceptable hardness (23,28) and a higher degree of conversion (8). This effect was also observed in the present study, including for dual cement specimens under direct light activation compared to negative control no photoactivated.

In this study the curing time of the resin cements was set at 30 seconds, but LED density power (40J/cm2) was 30% higher than the QTH (24J/cm2). However, there was no significant effect of the light sources on the DC values or on the KHN. The increase in irradiance does not necessarily lead to higher DC values, and the photoactivation directly on the polyester strip without interposed ceramic did not guarantee higher DC8 as also observed in this study. Evaluating the microhardness and indentation elastic modulus, density power did not influence the cement curing through ceramic up to 1.5 mm but suggested an increased exposure time for thicker ceramics (23).

For ARC, U200 and Veneer cements, KHN decreased with low translucency ceramics from 2mm to 4mm thickness. Kuguimiya *et al*. (29) observed higher nanohardness values for the ARC than for U200 when photoactivated under e.max Press restorations shade A2 and 2mm thickness. Flury *et al*. (23) have argued that Unicem 2 (similar to U200) is highly sensitive to the activation mode, which corroborated microhardness findings of this study. Archegas *et al*. (15) observed that for ARC and Veneer DC, the microhardness and elastic modulus were higher through translucent ceramic than opaque

Non-photoactivated self-adhesive RelyX U200 showed low DC and KHN values, which was previously observed (30) as dual cement requires photoactivation through ceramic restorations to achieve higher values of toughness (29). In areas where polymerization is inadequate, it is not expected that the improved physical and mechanical properties of the resin cements are achieved. No photoactivated ARC dual cement had KHN median similar to the cement photoactivated through low translucency ceramic of 3mm. It was found similar KHN for C&B and no photoactivated ARC, and higher KHN for ARC in the dual mode (24). In the present study, non-photoactivated U200 had KHN about seven times lower than when cured under low translucent 4mm thick ceramics. This finding reinforces the importance of curing light through the ceramic restorations, even a thicker, opaque or darker. Considering that in this study the ceramic shade did not influence the hardness of the cements evaluated, it can be inferred that for the chosen shade A, there was no influence of the ceramic saturation but only of its translucency.

From the results of this study, considering its limitations, to achieve higher values of microhardness and degree of conversion using lithium disilicate glass ceramic the following points are suggested: for high translucency ceramics up to 3mm thickness, A3 or a lighter shade, both RelyX ARC and RelyX U200 could be used, under LED curing (40J/cm2) or QTH (28J/cm2) for each side of the restoration. For high translucent ceramics darker than A3 and thickness above 4mm or low translucency above 3mm thickness, both dual-cured cements are indicated, but to increase light exposure time on each face should be considered.

## Conclusions

Despite the energy density emitted by the QTH lamp was 30% lower, the light sources did not influence the degree of conversion nor the values of Knoop microhardness. Shade, thickness and translucency of lithium disilicate glass ceramic affected the degree of conversion of resin cements RelyX ARC, U200 and Veneer. The Knoop microhardness of evaluated cements was influenced by ceramic thickness and translucency. Higher DC and KHN were achieved only for dual-cured cements photoactivated through 2mm-thick low translucent or 3mm-thick high translucent glass-ceramic.
